# TikTok’s *Falco tinnunculus*: Getting to Know Urban Wildlife through Social Media

**DOI:** 10.3390/ani13081292

**Published:** 2023-04-10

**Authors:** Duo Yin, Jiachun Chen, Quan Gao

**Affiliations:** 1 Centre for Human Geography and Urban Development, School of Geography and Remote Sensing, Guangzhou University, Guangzhou 510006, China; 2 School of Geography and Planning, Sun Yat-sen University, Guangzhou 510006, China; 3 China & Southern Marine Science and Engineering Guangdong Laboratory (Zhuhai), Zhuhai 519082, China

**Keywords:** social media, urban wildlife, knowledge production, representation, *Falco tinnunculus*

## Abstract

**Simple Summary:**

Wildlife adapting to urban environments has increased encounters between people and wild animals. The rise of social media, especially short video platforms, has provided more possibilities for people to experience human–wildlife encounters in virtual forms. In this case study, by sharing the co-habitation of humans and kestrels, the video producer performs a harmonious human–wildlife relationship on social media and fosters an intimate “companionship” for audiences. We argue that the knowledge production of wildlife from social media is a dynamic process co-created by multiple actors’ mutual connection and action. We also articulate the relationship between humans and wildlife in urban contexts; knowledge production and perception of these animals in social media demonstrate the unequal and unbalanced power relation held by different species.

**Abstract:**

Wildlife residing in cities has made encounters between humans and wild animals a common phenomenon. The perspective of the conflict-laden animal–human relationship has been over-emphasized by traditional media, which neglects the peaceful and harmonious daily encounters between residents and urban wildlife. This paper addresses the lacuna in extant literature by examining the virtual encounters between urban residents and wildlife on TikTok by sharing the living habits of *Falco tinnunculus*. Participatory observation, semi-structured interviews, and text analysis were adopted to explore the knowledge production process of urban wildlife as well as the emotional response of audiences. We found that displaying urban wildlife in short videos is a dynamic process involving the mutual participation of wildlife and humans. Meanwhile, audiences’ anthropocentric gaze of wildlife via TikTok attends to their desires for intimacy with nature and demonstrates the unequal and unbalanced power between wild animals and humans. These findings suggest that more efforts should be made to guide the public to pay attention to native urban wildlife species and to reflect upon the ethics and rationality of such unequal power relations between wild animals and humans.

## 1. Introduction

Urban space, albeit created by humans, has increasingly evolved into a complicated “more-than-human” space where human and non-human lives (wildlife) are intertwined in meaningful ways [1]. As urbanization and modernization progress, to offset the inherent conflicts created by the struggle between economic development, finite resources, and environmental conservation, green spaces in the form of community greenbelts, urban parks, and gardens have been created [2,3,4]. As the natural environment gets restored, some wildlife species are drawn to cities for permanent residency rather than as temporary stopovers or food scavenging places [5]. Besides traditional green spaces such as parks and gardens (e.g., [6]), wildlife is increasingly residing in residential balconies, gaps within buildings, and underground facilities where they nest, breed, and reproduce while sharing humans’ living spaces [7,8]. The intimate interaction between human and non-human lives in urban space has become a phenomenon that warrants in-depth investigation.

Technological advances of social media not only provide a nascent platform for people to receive information and engage in interpersonal communications, but also significantly influence the ways non-human lives are represented and comprehended by human beings [9]. Traditional mass media such as static printed words and still pictures no longer satisfy their audiences as people seek more excitement and variety through an emergent form of social media in the form of dynamic, short videos [10,11]. Amongst the diversity of everyday life videos shown on short video platforms, urban wildlife has attracted increasing attention, becoming an area of focus for content creators and users to present and comment upon [12].

The conflicts between humans and wildlife tend to attract mass media attention and, inevitably, academic scrutiny [13,14,15,16]. Folmer et al. found that contrary to dramatic conflicts between humans and large-sized predators, daily encounters with urban wildlife tend to occur in routine spaces such as gardens and parks [17]. The rise of social media, especially short video platforms, has provided more possibilities for these harmonious encounters. However, there is a paucity of research on adopting short videos as a tool to disseminate and produce knowledge of urban wildlife in routine daily life practices.

To address this lacuna, this paper considers the ubiquitous short-video-sharing platform TikTok as an empirical case study to examine: (1) the knowledge production process, general mechanism, and characteristics of wildlife videos in social media; (2) user perceptions of urban wildlife and the multi-species relationships behind it; (3) how do these wildlife videos affect the urban wildlife? How does the animal agency reflect and function during this process? Before presenting the empirical analysis, we first provide an overview of the theoretical framework of the relationship between wildlife and media.

## 2. Literature Review

Media coverage related to wildlife issues is usually in traditional mass media such as newspapers, television, and magazines, with the interviewed subjects being local authorities and subject matter experts focusing on sensational topics such as human–wildlife conflicts [18,19,20]. These reports usually present polarized views, dramatized and highly contentious content, which lacks in objectivity and scientific accuracy [21]. Gundrum et al. found that newspapers often act as the local authorities’ mouthpiece and portray different images of urban wildlife depending on governance needs [22]. These studies (e.g., [22,23]) demonstrate that the representation of wildlife by mass media is manipulated and is driven by an array of political, economic, and environmentally biased motivations. The everyday habits and characteristics of wildlife are selectively presented or ignored depending on political motivations.

Mass media is a one-way passive communication tool, with recipients unable to communicate with reporters or each other, resulting in disproportional influence and prejudice (e.g., [24,25]). These media reports are often highly biased towards sensational and violent human–wildlife conflicts involving large animals. Such negative and prejudiced reports may exacerbate human fears and spur irrational beliefs towards wildlife. Correspondingly, behavioral norms such as tolerance towards urban wildlife will decrease, as will support for urban wildlife conservation programs [26]. The unbalanced and biased mass media coverage of critical conservation issues may even cloud knowledge dissemination on the most pressing environmental issues (e.g., [27]).

Unlike mass media, much of the content released on social media originates from spontaneity in daily life. Capturing and introducing urban wildlife in short videos has also become one significant theme being produced on social media (e.g., [28]). Sharing and forwarding content takes on an influential role as content sharers’ emotions are passed onto others and result in mutual influence (e.g., [12]). The algorithmic recommendations of the video-sharing platforms also play an indispensable role in disseminating and popularizing content on social media [29,30]. Based on the user’s viewings, “likes” given, and video-forwarding behavior, the system will recommend similar videos to the user. Besides curated recommendations, social media also applies big data algorithms to the distribution process of popular videos so that trending and popular videos accumulate even more views [31]. This in turn further stimulates the production of related and trending videos [32]. In what follows, this article will examine how common urban wildlife living in harmony with urbanites is presented in social media, and analyze the knowledge production and the resultant influence on users, an area largely neglected by extant research.

## 3. Materials and Methods

TikTok (known as Douyin in China) is a short-video platform with a massive user base. It was established in 2016 by the Chinese technology giant, ByteDance. The app increased its popularity significantly in 2020, with 3 million downloads a day [33] and more than 1 billion active users as of 2021 [34]. To begin our search, we first used the keyword “urban wildlife” to retrieve related short videos on TikTok and then selected urban wildlife videos recorded by users in their everyday lives. The search results show that as of 15 August 2021, more than 1000 TikTok users have published videos on the theme of “urban wildlife encountered in daily life”. Most of these encounters took place on flowerpots outside the windows of residents’ homes or on balconies. The most common wildlife recorded were birds, including spotted dove (*Streptopelia chinensis*), blackbird (*Turdus merula*), grackle (*Gracula religiosa*), and magpies (*Pica pica*). We chose the most representative account, Qinhuangshanhai (秦皇山海), as our case study. This user account was created by Mr. Qin (anonymous name), residing in a coastal city (Qinhuangdao) in northern China. As of August 2021, Mr. Qin had created and uploaded 500 short observation videos detailing the daily lives of magpies and kestrels (*Falco tinnunculus*) nesting on his balcony for three consecutive years, attracting 36,000 followers and accumulating over 250,000 “likes” and 12,000 comments.

This study adopts the approach of participatory observation, semi-structured interviews, and content analysis. Observation was mainly used to explore the knowledge production process of urban wildlife, and we focused on and tracked Mr. Qin’s video content (such as bird life and bird behavior, video title, and background music, etc.), filming equipment evolution, live streams (time, duration, frequency), and his real-life interactions with his followers. To collect the large amount of data related to the videos, web crawlers, a program to automatically fetch the content of webpages [35], were adopted to crawl the titles, release dates, number of likes and comments, and comment contents of the short videos released by Mr. Qin in the past three years. After screening out irrelevant videos, 536 video recordings and 18,662 comments were retained for in-depth analysis. Concurrently, we also contacted Mr. Qin and conducted five semi-structured interviews with him by telephone over a total of nine hours. The interviews centered on themes such as his reasons for documenting magpies and kestrels, methods and equipment for video recording, daily activities on TikTok, interaction process with his followers, and his views and perspectives on urban wildlife.

The collected materials served multiple purposes. For each video, we watched and recorded the species (magpie or kestrel), breeding cycle (non-breeding season, pre-laying period, egg-laying period, incubation period, early nestling period, and late nestling period), and bird behaviors (a total of 21 kinds of bird behaviors, including eating, feeding, incubation, resting, etc.). To analyze the relationship between public preferences and the video producer’s operational decisions, we corresponded the number of comments and ‘likes’ for each video to different periods of the birds’ breeding cycle, and analyzed the differences in public attention to the birds over different time periods. At the same time, the behavior of the kestrel in the video was counted to analyze whether Mr. Qin’s selection of video content was influenced by audiences’ preferences. Moreover, we deployed text analysis to understand users’ sentiments towards Mr. Qin’s videos. First, we analyzed the word frequency of the data to make a preliminary opinion and summarize the main topics. Second, we read the corresponding specific text to identify specific views. Finally, we randomly selected a sample of 2000 comments out of 13,591 comments (after removing 4711 comments from Mr. Qin out of 18,662 comments) and counted the frequency of occurrence of the views of concern.

## 4. Results

### 4.1. Knowledge Production of Urban Wildlife on TikTok

In 2013, Mr. Qin found two magpies nesting in branches on his balcony. To record the breeding process of the magpies, Mr. Qin installed a surveillance camera next to the magpie nest in the second year (2014). In 2017, after several fierce fights and struggles, the magpies were chased away, and the victorious kestrels took over the nest. The kestrel is common throughout China and is one of the most common small birds of prey in eastern Chinese cities. Urban kestrels have a habit of seizing the nests of crows and magpies or building their nests in holes within human-made walls. By photographing the nest on his balcony, Mr. Qin successfully documented the breeding of two urban wildlife species: the magpie (2013–2016) and the kestrel (2017–present), which shared his family’s living space.

In February 2019, Mr. Qin created his account with the name of Qinhuangshanhai (秦皇山海) and began to upload short, themed videos on the daily life of urban wild birds, centered around his balcony’s bird nest. The videos’ content concentrated on magpies repairing nests, laying eggs, hatching, eating, and chick-rearing. One of the videos that broadcasted the sudden disappearance of the chick magpie’s parents and the resultant danger to the chick magpie won intense attention from TikTok users and became a top highly “liked” video of the day (exceeding 30,000 views). Through TikTok’s AI algorithms, this particular trending video was massively promoted on the platform, further amplifying the attention given to this particular video and Mr. Qin’s account, thereby helping Mr. Qin gain his earliest group of followers.

In his interactions with the TikTok users, Mr. Qin disclosed that kestrels had actually driven away the magpies shown in his short videos two years earlier and were now the new owners of the former magpies’ nest. This revelation sparked great curiosity amongst the users asking him to release the kestrels-related videos as soon as possible. However, Mr. Qin did not anticipate that the kestrels did not lay eggs in his family home’s nest that year but chose to breed in a hole within the wall of his neighbor’s house. In order not to disappoint his TikTok users and to sustain their interest in his account, Mr. Qin gave up his live-streaming plans and continued to upload pre-recorded videos from his accumulated video collection. Mr. Qin’s predicament highlights that unlike mass media’s pre-designed and pre-edited content, wildlife and its unpredictability have greater agency in the knowledge production for social media that prioritizes real-time dynamic updates. Humans do not have sole agency in determining how knowledge related to wildlife is produced. Wild animals’ preferences, decisions, and biological rhythms will also influence how humans broadcast and transmit short-term videos. Wild animals’ agency becomes apparent when kestrels choose whether to lay eggs in this nest, which changed the plans and decisions of the video producers when the magpies “abandoned” their two chicks, which led to speculation and heated discussion from audiences and the artificial feeding by the video producer. This brings more uncertainty, sometimes adding a dramatic plot, as well as sometimes going against the audience’s expectations, and becomes a key factor.

**Figure 1:**
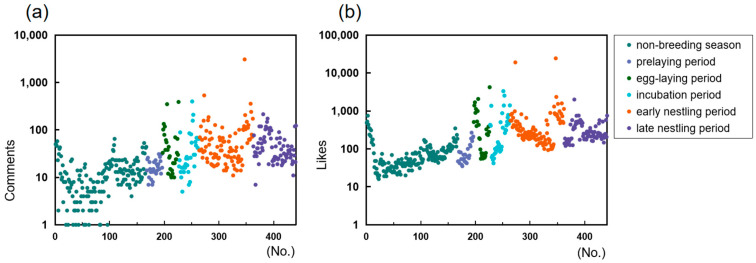
User attention based on different periods. (**a**) Number of comments on videos from different periods of kestrels. (**b**) Number of likes on videos from different periods of kestrels. Notes: Of the 536 related videos uploaded by Mr. Qin (as of 13 August 2021), more than 4/5 of them featured kestrels as the main character, and 441 of them could accurately determine the kestrel’s physiological cycle. We had reclassified these videos and plotted the number of comments and likes on the graph, with the X-axis representing the serial numbers of the classified videos. The figure was created by the authors using Excel.

**Figure 2:**
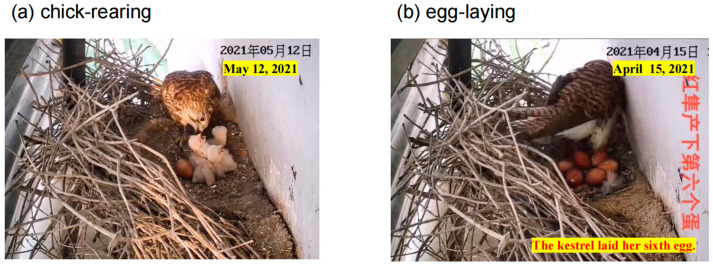
The mother kestrel’s chick-rearing (**a**) and egg-laying (**b**) behavior. Notes: The pictures were reproduced from the videos uploaded by Mr. Qin.

**Figure 3:**
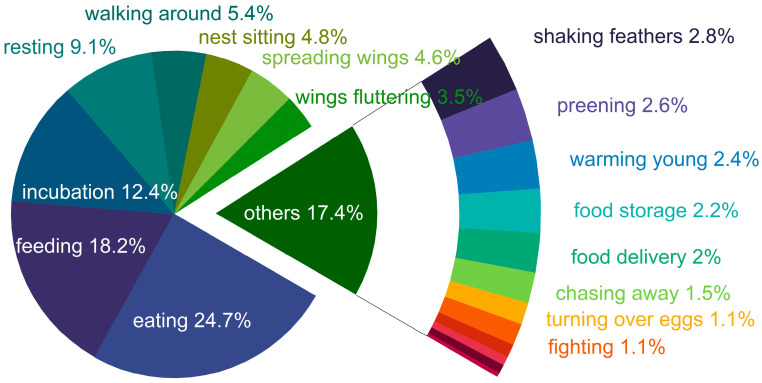
Proportion of activities portrayed in the TikTok Videos.

As Mr. Qin uploaded TikTok videos on the kestrels, users demonstrated more interest in these birds’ dynamic behavior (i.e., predating and chick-rearing) rather than their static nesting and hatching behavior. Based on our content analysis on user attention (Figure 1a,b), the kestrels received obviously less user attention in the non-breeding period. During its active breeding cycle, with the kestrels’ various behaviors of mating, hatching, and chick-rearing, there is a remarkable uptick in user attention and community discussion about the uploaded videos. Among them, the mother kestrel’s chick-rearing (Figure 2a) and egg-laying (Figure 2b) videos received the highest degree of user attention (3081 comments and 24,400 “likes”, 390 comments and 4222 “likes”, respectively). To attract even more user attention and “likes”, Mr. Qin released as many videos as possible about the kestrel’s chick-rearing through video editing and highlighted the kestrels’ diversified food sources and feeding movements in detail. Based on our data analysis (Figure 3), although Mr. Qin showcased many diverse behaviors and activities of the kestrels, and his videos included several rare and difficult-to-capture moments, such as the kestrels mating or fighting as well as their chicks’ defecation process (Figure S1), he still chose to prioritize the videos on their eating and feeding processes as the central theme in his account because these videos tend to attract the most user attention and “likes”. Therefore, users’ preferences can dictate the content production in these urban wildlife videos and as this user-preferred content gains momentum through more “likes” and sharing, TikTok’s data algorithms will pick up these popular videos and recommend them to even more people who may be interested in these videos. This, in turn, propels the creation and expands the growth of virtual communities interested in urban wildlife and their daily routines.

As Mr. Qin engaged in more interaction with fellow TikTok users, he started his own fact-finding process on kestrels so that he would have the factual and scientific knowledge to answer their questions. He educated the users on distinguishing between male and female kestrels, their breeding cycles, and the food sources of young chicks. However, mindful of user preferences, Mr. Qin was still purposeful in his selection of the kestrels’ daily routines; therefore, the knowledge production of these kestrels was neither objective nor holistic. Although social media allows instant interactions among users and more knowledge sharing, fact-finding, and verification among its content producers and users, the wildlife discourse produced does not necessarily become more scientific or objective in presentation.

Besides capturing videos using cameras, the knowledge production of wildlife in social media also directly impacts wildlife. In June 2019, Mr. Qin found three chick kestrels learning to fly on the grass and decided to put them into the empty nest left by previous kestrels in his house in the name of “rescuing them”. Mr. Qin’s “rescue” successfully attracted the mother kestrel of the chick kestrels to come and feed them. Mr. Qin then proceeded to create and upload 20 highly successful themed videos, winning many “likes” for these videos. From this encounter, it is clear that social media interactions between content producers and users go beyond encouraging virtual discussion and dissemination of urban wildlife content online, but exerts an actual influence on urban wildlife survival in cities.

In 2020 and 2021, the kestrels again chose Mr. Qin’s balcony nest and proceeded to breed there. Resultantly, Mr. Qin could create and upload actual-day videos and directly socialize with his users in real time. To optimize his video quality, he upgraded his filming and surveillance equipment to capture the kestrels’ living habits and routines thoroughly from multiple angles and panoramic displays. He also gradually developed a routine schedule for video uploads. Mr. Qin also interacts with his users in his virtual live-streaming room, and discusses and answers users’ questions on his latest video’s contents, thereby building and enhancing a sense of community among his users, leading to stronger community cohesion. From the first chick hatching to the last chick leaving the nest, Mr. Qin led users to observe and discuss the lives of his wildlife neighbors, therefore creating a bridge between the virtual and the actual via participatory “companionship”. This “companionship” portrays a form of daily, relaxing, and harmonious routine human–urban wildlife relationships unladen with stress or anxiety.

### 4.2. Users’ Perception of Urban Wildlife through TikTok

**Figure 4:**
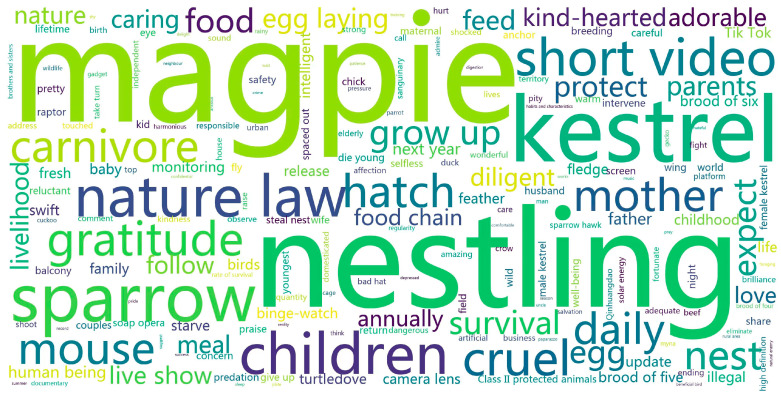
Perceptive word cloud map.

A word frequency analysis was conducted to study the influence of these TikTok wildlife videos on their users. From the word cloud analysis (Figure 4) and the word frequency count (Table S1), it can be seen that user perception of urban wildlife has evolved around the intertwined relationships between wild animals and non-human elements (i.e., magpie, kestrel, sparrow, mouse, prey, predation, food chain, lay eggs, hatch, survival, occupy, and so on). There was also interest in wildlife conservation (i.e., protect, nature, laws of nature, illegal, second-class protected animals, intervention), sentiments towards wildlife (i.e., gratitude, expect, cruel, and selfless), how social media was represented (i.e., short video, live show, camera lens, etc.) and post-video watching behavior (i.e., applaud, moved, hope, release, etc.). Next, we integrated our empirical findings and textual data to further explore social media users’ perspectives on urban wildlife.

Firstly, urbanites are unfamiliar with their wildlife co-inhabitants and generally possess minimal knowledge of their characteristics or habits. By examining users’ comments on the videos produced by Qinhuangshanhai, this paper finds that alongside China’s policies on ecological conservation, there is an increase in wildlife population and their residency in urban cities (mentioned by 1.45% of the comments). Nonetheless, online comments can also reveal the minimal knowledge and lack of fundamental understanding of urban wildlife by the general Chinese public. Many netizens had seen the wild birds featured in Mr. Qin’s videos before in their daily life; however, they were unable to identify either the species or their habits (mentioned by 14.49% of the comments). Some users confused magpies with crows and expressed doubts, “Are these really magpies? They look like crows” (comment from the user Taier (太二)).

Secondly, there exists a disparity in the acceptance of factual scientific knowledge about urban wildlife. Many users cannot fully accept the realistic behaviors and images presented by Mr. Qin in his videos on kestrels’ chick-rearing and predatory practices. For example, on 12 May 2021, Mr. Qin uploaded a detailed video on the chick-rearing practices of kestrels. The video showed the mother kestrel ripping off a mouse’s head with her sharp, pointed beak and popping out a sparrow’s eyes to open its skull. Many users expressed distress at such a grisly sight (mentioned by 21.68% of the comments of this video) and lamented, “It’s too cruel” (comment by user Hui (辉)) and “I cannot continue watching” (comment from the user Gezhemenger (歌者梦儿)). Compared to the wildlife’s actual biological habits, users mostly prefer images congruent with their personal interests. For example, traditional Chinese culture profoundly impacts the Chinese public’s perception of wildlife. In Chinese culture, many wild animals are considered symbolic and represent luck and auspiciousness. Seeing them or sharing their living space implies that the receiver and their families will have good luck. Therefore, many users would convey their prayers and wishes in the video of the magpies’ nest uploaded by Mr. Qin (mentioned by 1.11% of the comments).

Finally, users tend to display polarized attitudes towards wildlife: strong anthropocentrism (7.73% of comments) and weak anthropocentrism (29.95% of comments). Users with strong anthropocentric attitudes tend to disregard wildlife and harbor ill intentions towards them. They may also objectify wildlife as entertainment tools to satisfy their own needs, disregarding animal rights and welfare. For example, on Mr. Qin’s account, there were endless recurring callous comments related to the sale of the kestrels, such as “sell one to me”, “how much to buy a kestrel, give me a price”. Some users also attempted to sabotage the incubation of the kestrels by instigating Mr. Qin to swap the kestrels’ eggs for chicken eggs for the kestrels to try to hatch, claiming this would increase the entertainment value for his followers.

Users with weak anthropocentrism tend to perceive the TikTok videos as a way of companionship and caring towards the kestrels. Some users have developed strong emotions towards these kestrels. One user proclaimed herself as a loyal follower as she commented that she has been following the process from “egg laying to shell breaking to nest leaving” (comment from the user Kaixinxiaomai (开心小麦)). Another user said that “watching the kestrels is like watching a TV drama where there are moments of happiness, worries and more often than not, touching moments” (comment from the user Benben (笨笨)). Users often used personification to relate the kestrels as mortal subjects with human emotions such as happiness, sadness, and individual preferences (19.81% of comments). Some exclaimed that “animals also have rich emotions. May their children grow up happily and healthily just like human children!” (comment from the user Yuanyezihua (原野之花)).

However, these users’ care and concern for the kestrels are not built on moral regard for animal rights but more out of their innate need for entertainment, stress alleviation, and the desire to feel close to nature (9.14% of comments). When the kestrel chicks grew up and left the nest, one commented that “This is not what I want to see, an empty nest! If I could, I would rather lock them up so that I can continue to see them” (comment from the user Yaoshi (钥匙)). Another threatened Mr. Qin to “think of ways to retain the kestrels, or we will cancel our subscription to your account” (comment from the user Heermengdepenfa (荷尔蒙的喷发)). When the youngest chick kestrel died unexpectedly in 2021, many users lamented that they no longer wanted to follow Mr. Qin’s videos now that the chick had died. Others blamed Mr. Qin for not using artificial resuscitation to save the chick from death. These users’ views are consistent with animals’ ethics and welfare theories. They do not reject the use or sacrifice of animals for human interests, nor do they advocate abstract equal rights between animals and human beings. From these Tiktok users’ perspective, the relationship between humans and wild animals remains unequal, where humans still objectify wildlife to fulfil human needs and desires.

**Figure 5:**
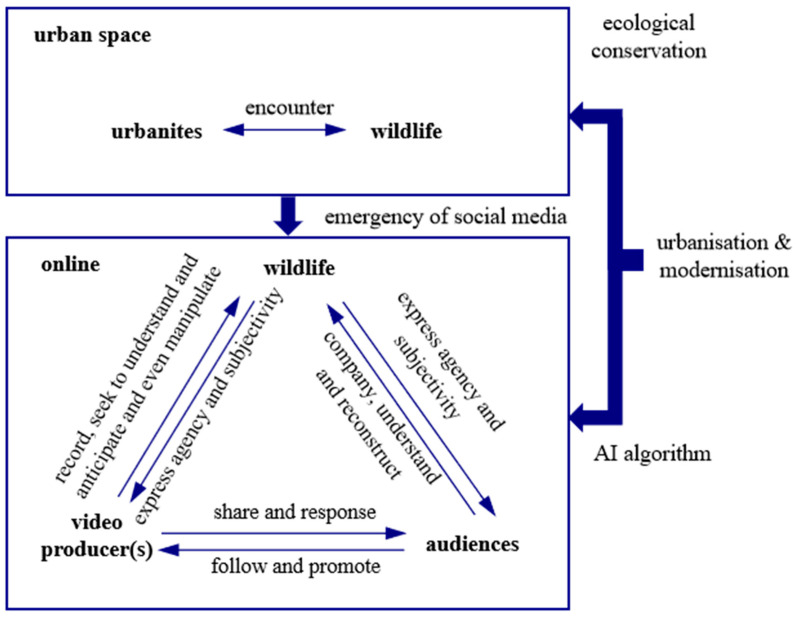
The general pattern of human–urban wildlife relationship in social media.

In this case study, accordingly, we propose the general pattern of human–urban wildlife relationships in social media (as shown in Figure 5). The video producer, influenced by the audiences’ interests and preferences and wildlife initiative, constantly adjusts and changes his practice and decision-making. The audiences constantly input, output, and reconstruct the information about wildlife in the interaction with the video producer, and promote the wildlife video operations of the video producer through feedback and reflection. Wild animals’ practice is often independent of human’s understanding and anticipation. The AI algorithm also becomes a key factor in influencing knowledge production with its calculative recommendations in amplifying attention on the highlights of the wildlife videos.

## 5. Discussion

The limitation of this study was that we focused on one case study, which may limit the generalization of the research findings. That is, our research focus centered on the specific birds, platform users, and followers around one TikTok video producer. The general pattern of human–urban wildlife relationships in social media based on this study needs validation for other species and in other social media platform, as effects generated from human–urban wildlife encounters and interactions in social media varies from different wildlife due to the heterogeneity of species. Therefore, we call for more research to give wider attention to different urban wildlife species and social media platforms.

This study contributes to the existing literature in several aspects. Our research proves that unlike mass media, which is unidirectional and single-step [15], knowledge sharing on the human–wildlife relationship in social media is more mutual, immediate, and interactive among the users. In contrast, consistent with the mass media [18], social media still present biased and constructed information on wildlife. With the purpose of sustaining user attention as much as possible, different biological characteristics and daily routines of wild animals are strongly or weakly emphasized, and their behaviors are given personification, with sudden, abrupt human interruption (if it is a trending users’ request). The real-time webcam of wildlife such as nesting falcons and hawks is also popular, as it is supplemented with scientific facts being added to the footage and spaces, allowing viewers to leave comments. Compared with TikTok videos, there might be no deliberate operations by video producers guiding users’ perception of wildlife, and thus the audiences are free to comment and make sense of the wildlife in their own preferred ways. Such videos (whether in social media or other forms) should therefore be critically examined by future research in terms of their effects on the knowledge production of wildlife and their influences on public awareness and attitudes towards wildlife.

Secondly, this paper sheds light on the everyday relationship between humans and urban wildlife by exploring the virtual encounter between humans and ordinary urban wildlife in daily life. Additionally, the online community mesmerized by the TikTok videos reflects a contradictory phenomenon: although people routinely ignore the native and common wildlife species around them, they also show strong interest in these species in the internet platform. This paper argues that ecological and biodiversity protection and related regulatory bodies could play a guiding role in educating the public to pay attention to native urban wildlife species, attracting and possibly motivating urbanites to participate in urban wildlife conservation. In addition, when wildlife “trespass” and reside in the gaps of highways, in the wall holes of large commercial centers, and even heritage and historical buildings under conservation status, the human–wildlife relationship has expanded beyond the urban–ecological environment into larger implications within the broader economic and cultural context of the city.

This paper also articulates the relationship between humans and wildlife in urban contexts—knowledge production and perception of these animals in social media demonstrate the unequal and unbalanced power held by different species. The rapid advancement of urbanization and modernization has accelerated urbanites’ desire to feel close to nature. In wildlife-themed tourist attractions, wildlife may be considered well-treated and cared for following the legal and political regulations of modern civilization; however, there still exists a human propensity for violence, dominance, and threats to these animals [36]. Even though the human–animal encounter is virtual in the short-video social media platforms and seem to present no threat to the well-being of the presented wildlife, the community’s displayed behavioral norms and attitudes still reflect that the relationship is human-centered and contrived to fulfil human interests and needs. The ethics and rationality of such a relationship need to be reflected upon, and animals as a moral and political subject could also be included in the politics and regulations of broader environmental communication and urban management practices.

Finally, this paper demonstrates the lack of public knowledge and awareness of wildlife conservation in an urban context. Some online questionnaire-based surveys across China show that over 90% of respondents (who we believe were mostly urban dwellers) were in favor of a complete ban on eating and trading wildlife after the COVID-19 outbreak, and that the vast majority of respondents had a willingness to learn about wildlife knowledge and laws. However, they acknowledged their knowledge of wildlife was insufficient [37]. The findings of this paper are consistent with the fact that public knowledge and awareness of wildlife conservation should be further enhanced.

## 6. Conclusions

This paper focuses on the common, coincidental, and even easily overlooked everyday life encounters between urban residents and the wildlife co-sharing their living space, and examines such encounters by analyzing short videos (an important emergent form of social media) and the perception and attitude of short-video media users towards these wildlife encounters. We found that in this process, the content producers, the users who follow the videos, and the displayed wildlife are in continuous interaction, enriching the viewers’ emotions, knowledge, and actions. Meanwhile, both content producers and their users seek to understand and anticipate wildlife behavior and “performance” from a perspective congruent with their personal interests and motivations. Such a purposeful social construction of wildlife demonstrates the unequal power relations between wild animals and humans. In general, we believe that such videos should be viewed critically. This shows the everyday relationship between humans and urban wildlife beyond conflict and tension by capturing the scenarios of urban wildlife. In contrast, the knowledge of wildlife received by users is neither objective nor holistic, as the knowledge of urban wildlife is subject to discursive practices rather than scientific knowledge per se. Our findings suggest that more efforts should be made to guide the public to pay attention to native urban wildlife species and to reflect upon the ethics and rationality of such unequal power relations between wild animals and humans.

## Data Availability

Restrictions apply to the availability of these data. Data was obtained from TikTok and are available from the authors with the permission of TikTok and Qinhuangshanhai (秦皇山海).

## Supplementary Materials

The following supporting information can be downloaded at: https://www.mdpi.com/article/10.3390/ani13081292/s1, Figure S1: Examples of kestrel behavior; Table S1: The top 105 high-frequency words.
