# Supplementary material for: TikTok’s Falco tinnunculus: Getting to Know Urban Wildlife through Social Media
Source: Animals (Basel). 2023 Apr 10;13(8):1292. doi: 10.3390/ani13081292 (PMC10135062; doi:10.3390/ani13081292)
Supplement: Supplementary file 1 [file animals-13-01292-s001.zip › animals-2260659-supplementary.pdf]

Supplementary Materials

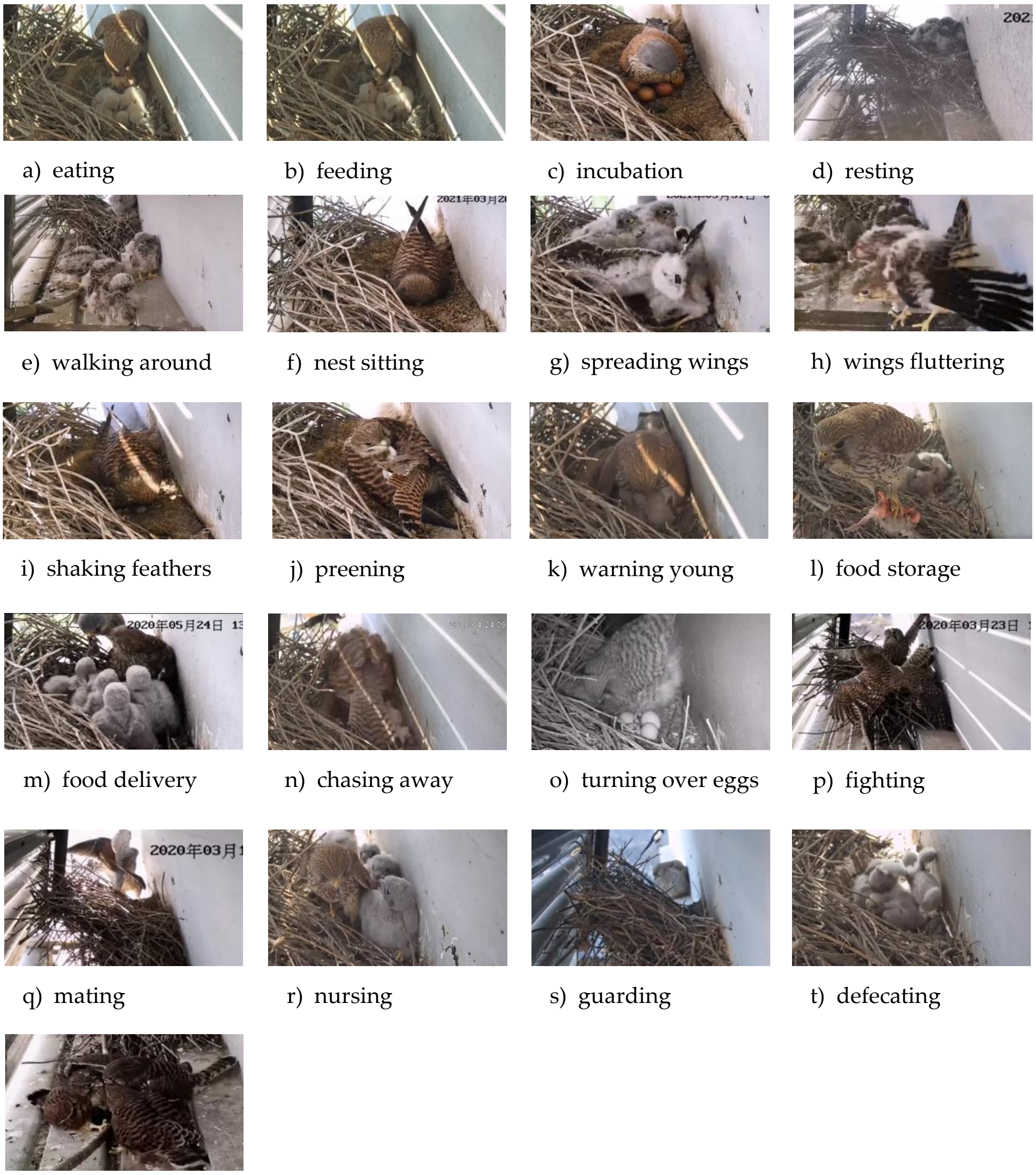

a) eating
b) feeding
c) incubation
d) resting
e) walking around
f) nest sitting
g) spreading wings
h) wings fluttering
i) shaking feathers
j) preening
k) warning young
l) food storage
m) food delivery
n) chasing away
o) turning over eggs
p) fighting
q) mating
r) nursing
s) guarding
t) defecating
u) competing for food

Figure S1. Examples of kestrel behaviour.

Notes: We labeled the birds' behaviour shown in each video. The example pictures are reproduced from the videos uploaded by Mr.Qin.

Table S1. The top 105 high-frequency words

Rank	Word	Frequency	Rank	Word	Frequency	Rank	Word	Frequency
1	magpie	937	36	annually	122	71	world	60
2	nestling	754	37	human being	122	72	balcony	59
3	kestrel	717	38	father	121	73	eye	59
4	sparrow	626	39	next year	118	74	couples	59
5	nature law	444	40	monitoring	117	75	maternal	59
6	children	441	41	illegal	117	76	pretty	56
7	short video	416	42	starve	116	77	youngest	55
8	cruel	398	43	swift	109	78	well-being	55
9	gratitude	380	44	update	109	79	chick	55
10	hatch	358	45	turtledove	104	80	selfless	55
11	mouse	354	46	fresh	102	81	wife	54
12	mother	353	47	feather	101	82	wing	53
13	carnivore	339	48	birds	94	83	concern	51
14	daily	316	49	brood of five	92	84	reluctant	51
15	nest	290	50	camera lens	92	85	warm	51
16	grow up	279	51	release	90	86	soap opera	50
17	expect	251	52	intelligent	90	87	anchor	48
18	food	250	53	binge-watch	89	88	intervene	48
19	survival	246	54	fledge	88	89	Female kestrel	47
20	egg	234	55	baby	87	90	screen	46
21	protect	227	56	childhood	86	91	male kestrel	45
22	egg laying	219	57	family	80	92	kid	44
23	feed	209	58	Tik Tok	79	93	wild	43
24	parents	205	59	brood of six	76	94	husband	43
25	caring	181	60	life	74	95	field	42
26	meal	176	61	Class II protected animals	71	96	careful	42
27	kind-hearted	171	62	praise	70	97	night	42
28	diligent	164	63	fly	70	98	spaced out	41
29	follow	152	64	steal nest	68	99	die young	40
30	live show	148	65	predation	66	100	safety	40
31	adorable	148	66	raptor	65	71	world	60
32	nature	147	67	return	64			
33	livelihood	136	68	share	63			
34	food chain	135	69	lifetime	62			
35	love	123	70	breeding	61			
